# Effects of a textile azo dye on mortality, regeneration, and reproductive performance of the planarian, *Girardia tigrina*

**DOI:** 10.1186/s12302-014-0022-5

**Published:** 2014-08-21

**Authors:** Alyson Rogério Ribeiro, Gisela de Aragão Umbuzeiro

**Affiliations:** School of Technology, State University of Campinas - UNICAMP, Paschoal Marmo Street 1888, Limeira, SP 13484-332 Brazil

**Keywords:** Regeneration, Planarians, Reproductive performance, Azo dye, Disperse red 1, *Girardia tigrina*

## Abstract

**Background:**

Many dyes can be considered emerging contaminants. The most widely used dyes belong to the class of azo compounds, some of which are known to have toxic and genotoxic properties. They are used in great quantities in textile activities and are of environmental concern because of their potential discharge in water. Planarians have been successfully used as test organisms in hazard evaluation of different chemicals, and we demonstrate the suitability of *Girardia tigrina* for laboratory testing. The aim of this work was to evaluate the suitability of the planarian species *G. tigrina* to assess the ability of the azo textile dye disperse red 1 to cause acute toxicity and adverse effects in the regeneration and reproduction of newborn and adult specimens.

**Results:**

Disperse red 1 presented a median LC_50_ of 75 and 152 mg/L, respectively, for newborns and adults of *G. tigrina*, showing that newborns are twice as susceptible to the dye. Uncoordinated movements, irregular twists, colored skin, increased mucous production, and regenerative delays were observed after dye exposure at sub-lethal concentrations.

**Conclusions:**

A no-observed-adverse-effect concentration (NOAEC) of 0.1 mg/L could be determined for disperse red 1 based on the fecundity test. Zinc seems to be a suitable positive control for monitoring the sensitivity in *G. tigrina* tests within only 24 h of exposure. This study demonstrates the applicability of *G. tigrina* tests in the hazard evaluation of water contaminants, such as azo dyes.

## Background

Synthetic dyes can be considered emerging contaminants because they are potentially toxic and have been found in the aquatic environment and there are no regulations stating the maximum allowable concentrations in water to ensure the protection of aquatic biota and human health. The world production of key dyes is estimated to be more than 10 tons per year [1]. The most widely used dyes belong to the class of azo compounds derived from certain aromatic amines and are used in products such as textiles, foodstuffs, cosmetics, house products, paints, and inks. This is significant because some of these dyes are known to have potential toxic and genotoxic properties [2,3]. The use of azo dyes to the color of textiles is of considerable interest, as this can raise environmental concerns because of the high volume of water involved in the dyeing process. When a dye is used in this process, a portion does not undergo bonding to the fibers thus remaining in the water bath [4]. As a consequence, high volumes of wastewaters containing dyes and related auxiliaries are produced and may be released into the environment. For instance, for 10 kg of polyester fabric, 100 g of disperse red 1 will be dissolved in 200 L of water, of which are round 1% of the dye will remain in the water bath at the end of the process. Hence, around 200 L of wastewater containing 1 g of the dye will be generated. It is also known that conventional treatments involving aerobic lagoons or activated sludge are not efficient in the removal or biological degradation of these dyes [5] and therefore, the dyes are still present in the final effluent or in the sludge of the treatment plant [6,7]. Disperse dyes used to dye synthetic fibers are generally sparingly water-soluble compounds, but they can be found in the water column, because of their commercial formulation that includes surfactants needed for the dyeing process. Some disperse dyes have been found in the aquatic environment [8], and their presence was related to the observed mutagenic activity of the water and sediments [6,7,9].

Recently, Ferraz et al. [10] showed that the textile dye disperse red 1 ((*N*-ethyl-*N*-(2-hydroxyethyl)-4-(4-nitrophenylazo) aniline) is highly toxic to aquatic invertebrates, in addition to being mutagenic. Vacchi et al. [11] studied a commercial disperse red 1 dye product and found that the ecotoxicity of the commercial preparation was similar to the dye itself. The median effective concentration (EC_50_) for *Daphnia* was 0.1 mg/L; therefore, this dye can be classified as highly toxic to aquatic organisms according to the Globally Harmonized System of Classification and Labelling of Chemicals (GHS) [12]. More recently, studies have been performed to estimate the concentrations of dyes in surface waters [13], Zocollo et al.'s submitted article. The authors determined the concentrations of disperse azo dyes in river and effluent samples collected in Brazil and showed that they were present in the levels of nanograms to micrograms per liter. However, more studies are required to estimate the concentration levels in the aquatic environment, especially in countries where dyeing activities are more intense, like in India, China, and Brazil.

Planarians have been used as test organisms in the hazard evaluation of different chemicals using different endpoints such as mortality, regeneration, micronucleus (MN) frequency, and enzymatic activity [14-21]. Planarians generally reproduce by transverse fission, but some species, like *Girardia tigrina* [22], also have hermaphroditic sexual organs and can generate cocoons of fertilized eggs [23]. Therefore, reproduction impairment can also be used as an endpoint in ecotoxicity tests with planarians [20,22].

Because of its ability to regenerate [24,25], planarians have been used to verify if chemicals can interfere with this process [17,18,26-30]. A small body fragment can generate an intact planarian due the presence of totipotent stem cells called neoblasts, which migrate from the parenchymal tissue to the injured site and differentiate by mitosis to other planarian cell types [31,32].

There have been a number of publications that examined the response of planarians to chemical exposures using molecular and enzymatic approaches [21,33-35]. We focused our study towards three different low-cost and environmentally relevant endpoints: mortality, regeneration, and reproduction. The aim of this work was to evaluate the suitability of the planarian species *G. tigrina* as a test organism to assess the acute toxicity of the azo textile dye, disperse red 1, to newborns and adults and to verify its ability to cause adverse effects on regeneration and cocoon production in the exposed animals. Disperse red 1 was selected because it has been found in river waters that receive textile effluents in the region of Americana, São Paulo, Brazil (Zocolo et al.'s submitted article).

## Results and discussion

*G. tigrina* showed an acceptable sensitivity to ZnSO_4_, with a 24-h median lethal concentration of LC_50_ of 1.6 ± 0.2 mg/L expressed in Zn^2+^ and a variation coefficient of 12% in seven replicate acute newborn toxicity tests (Table 1). Chromium salts have previously been used to monitor sensitivity of laboratorial culture [15,36,37]. We also used chromium for this purpose but zinc provided better repeatability and faster responses (24 h) (Table 1). Although the mean LC_50_ values after 48 and 96 h for chromium are similar to those reported by Preza and Smith [15], the coefficient of variation (66%) in our study was higher than obtained by those authors (22%). This higher variation could be explained by the higher instability of chromium in solutions compared to zinc or by response differences between the planarian populations.

**Table 1:** **Mean lethal concentrations (50%) (LC**_**50**_ **) of chromium and zinc in newborn** ***G. tigrina*** **acute tests for sensitivity assessment**

	**LC**_**50**_ **(mg/L)**							
**Test**	**Cr** ^**6+**^				**Zn** ^**2+**^			
	**24 h**	**48 h**	**72 h**	**96 h**	**24 h**	**48 h**	**72 h**	**96 h**
I	13	11	8	7	2	2	2	2
II	11	11	11	7	1.6	1.6	1.6	1.6
III	*	27	20	11	1.6	1.6	1.6	1.6
IV	*	29	18	15	1.6	1.6	1.6	1.6
V	18	8	5	3	1.3	1.3	1.3	1.3
VI	15	10	5	4	1.6	1.6	1.6	1.6
VII	14	5	2	5	1.7	1.7	1.7	1.7
Mean	14.2	14.4	9.8	7	1.6	1.6	1.6	1.6
SD^a^	2.5	9.5	6.8	4.6	0.2	0.2	0.2	0.2
CV (%)^b^	18	66	69	66	12	12	12	12

Asterisk indicates LC_50_, not calculable for the statistical method employed. ^a^SD standard deviation of the mean. ^b^Coefficient of variation. The tests indicated as I, II, III, IV, V, VI, and VII correspond to independent experiments.

The LC_50_ values for disperse red 1 dye obtained with newborns and adults are presented in Table 2, and as expected, toxicity increased with exposure time. Three independent tests were performed, and the coefficients of variation of the means were below 17%, which was considered acceptable for ecotoxicological assays according to the recommendation of Environment Canada [38]. No mortality was observed when adults were exposed during the first 24 h but several animals showed uncoordinated movements and increased mucus production at all tested concentrations. Mean 96-h LC_50_ values of 75 ± 7.2 mg/L (*n* = 3) and 152 ± 5.8 (*n* = 3) mg/L were obtained for newborns and adults, respectively (Table 2). These results confirmed the findings of Preza and Smith [15] which showed that newborns were more sensitive than adults. Knakievicz and Ferreira [20] attributed this age response to different body surface/volume ratios of newborn organisms.

**Table 2:** **Mean lethal concentrations (50%) (LC**_**50**_ **) of commercial disperse red 1 in newborn and adult** ***G. tigrina*** **tests**

	**LC**_**50**_ **(mg/L)**							
**Test**	**Newborn**				**Adult**			
	**24 h**	**48 h**	**72 h**	**96 h**	**24 h**	**48 h**	**72 h**	**96 h**
I	127	127	74	67	*	201	179	159
II	120	91	79	79	*	207	156	148
III	131	111	87	80	*	198	162	150
Mean	126	110	80	75		202	167	152
SD^a^	5.6	18	6.5	7.2		4.6	12	5.8
CV (%)^b^	4.4	16.4	8.1	9.6		2.2	7.2	3.8

Asterisk indicates no mortality was observed. ^a^SD standard deviation of the mean. ^b^Coefficient of variation. The tests indicate as I, II, and III correspond to independent assays.

Ecotoxicological data of azo textile dyes are scarce in the literature. Ferraz et al. [10] tested disperse red 1 (95% purity) and obtained an EC_50_ of 0.13 mg/L in a *Daphnia similis* acute toxicity test. Recently, the same commercial product that was analyzed in this study was used in acute toxicity tests with *D. similis* and *Hydra attenuata* [11]. The authors showed that the surfactant and other impurities present in the commercial dye did not influence the observed toxicity, at least for *Daphnia*. The observed effects were related to the main dye, which represents 60% of the commercial product. In that study, it was not possible to verify if the acute toxicity observed for Hydra was related also to the main dye, but Hydra was 15 times less sensitive than Daphnia to the commercial dye [11].

Aggregation and precipitation of the dye were observed at concentrations higher than 50 mg/L after the first 24 h of exposure. This is expected because the commercial dye contains surfactants. Therefore, when the concentration of the disperse dye increases, they can form aggregates and settle on the bottom. This behavior is also expected to occur in the aquatic systems [39]. Because planarians slide on the surface of the testing containers [40], the animals were exposed to the precipitated/agglomerated dye in addition to the dye remaining in the solution. This natural surface-contact behavior of the planarians is an important consideration when assessing the toxicity of compounds that are sparingly soluble in water.

**Figure 1:**
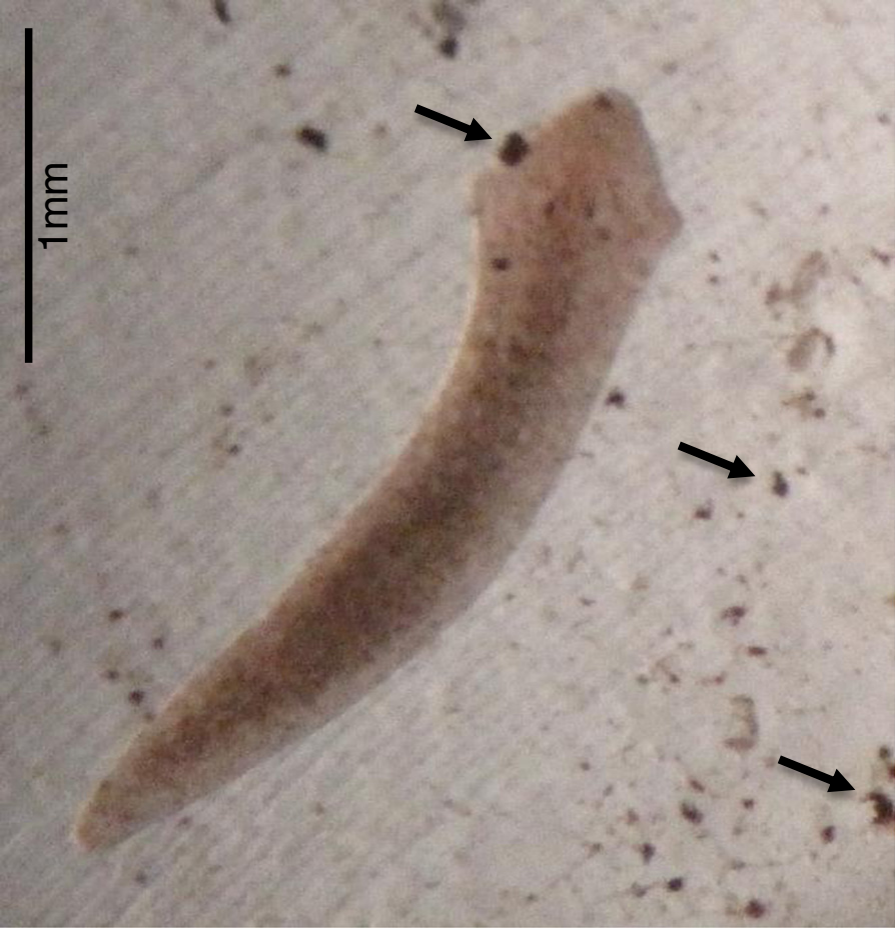
**Picture of a newborn of** ***G. tigrina*** **after 96-h exposure to 10 mg/L of commercial disperse red 1 dye** ***.*** Red-colored skin can be observed. Arrows indicate the precipitation and agglomeration of the dye. The image is 50 times increased.

After 24 h of exposure in the acute toxicity tests, newborns (Figure 1) and adults (not shown) exposed to concentrations higher than 10 mg/L showed red-colored skin, especially at the encephalic region. This could be explained by dermal or cilia dye adsorption during locomotion and/or skin respiration. The exposed organisms showed uncoordinated movements, irregular twists and increased mucous production after the first 24-h exposure. The mucous resulted in dye precipitation and aggregation around the animal bodies, forming what appeared to be a body capsule, which was abandoned when the planarians started to move. Planarian behavioral effects, such as increased mucus production, irregular twisting, and body contractions, have previously been associated with animal responses to toxicants [26,41,42].

**Figure 2:**
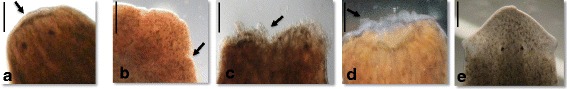
**Morphological effects of different concentrations of commercial disperse red 1 dye in** ***G. tigrina*** **. (a)** Individual without full body cicatrization, showing eyespots and no auricles (phase z) - after 120-h exposure at 200 mg/L. **(b)** Late development of the auricle (phase d) after 120 h at 50 mg/L. **(c, d)** Individuals with delayed and abnormal cicatrization (phase t) - after 120 h at 200 mg/L. **(e)** A fully developed planarian with no effect (negative control). Arrows are guides to the eye and the bar on the left of each figure represent 1 mm.

In the regeneration assay, the first changes were observed after 24 h at the highest concentration tested, 200 mg/L. After 120 h, 95% of control planarians fully regenerated (see Figure 2e). The no-observed-adverse-effect concentration (NOAEC) for the dye was 10 mg/L and the lowest-observed-adverse-effect concentration (LOAEC) was 50 mg/L (Table 2). The regenerated planarians showed also red-colored skin, behavioral changes, and increased mucous production at concentrations higher than 10 mg/L. Regenerative delay started to occur at 50 mg/L, while at the highest concentration (200 mg/L), eight of the 20 organisms died within 96 h of exposure, and the ones that survived presented severe regenerative delay after 120 h of exposure (Table 3) with parenchymal loss due to lack of wound cicatrization (see Figure 2c,d). When cicatrization occurred, some delays in posterior regenerative stages were noticed. In seven animals the auricle development was inhibited or delayed (see Figure 2a and 2b). For successful regeneration of the lost body parts, planarians need an intact nervous system to control the migration of the normal neoblasts [43,44] that may have been affected by the dye. Also, the mutagenic properties of the dye [10,11] could affect the normal mitotic process required for the cell replication, which is needed for regeneration. The planarian regeneration assays are useful for analyzing the effects of chemicals on cell differentiation and molecular organization [45]. Thus the migration and proliferation of neoblasts as well as other steps required to *G. tigrina* regeneration may have been affected by the azo dye.

**Table 3:** **Regenerative results for** ***G. tigrina*** **exposed to different concentrations of the commercial disperse red 1**

**Exposure time (h)**	**Planarians/regeneration steps (mg/L)**					
	**Control**	**10**	**50**	**100**	**150**	**200**
12	20^a^ (a)^b^	20(a)	20(a)	20(a)	20(a)	20(a)
24	20(b)	20(b)	20(b)	20(b)	20(b)	3(t) 17(b)
48	20(c)	20(c)	20(c)	20(c)	20(c)	3(t) 17(c)
72	20(de)	20(de)	20(de)	2(c) 18(de)	2(c) 18(de)	3(t) 17(de)
96	20(f)	20(f)	3(d) 17(f)	5(d) 15(f)	2(d) 18(f)	3(t) 9(d) 8(f)
120	1(f) 19(g)	20(g)	2(f) 18(g)	5(f) 15(g)	5(f) 15 (g)	8 dead 3(t) 3(z) 4(d) 2(g)
Pd^c^_120h_		−1	1	4	4	9

^a^Number of animals. ^b^Regeneration steps: (a) decapitated bodies, (b) beginning of blastema formation, (c) regular blastema, (d) beginning of auricle formation, (e) beginning of eyespots formation, (f) auricle and eyespots clearly defined, (g) full head formation, (t) body section without cicatrization, (z) without body cicatrization, with eyespots, and without auricle. ^c^Pd is the number of planarians with delayed regeneration subtracted from the number of delayed planarians in the control, Pd greater or equal to 1 is considered as adverse, for more information see ‘Methods’ section.

**Figure 3:**
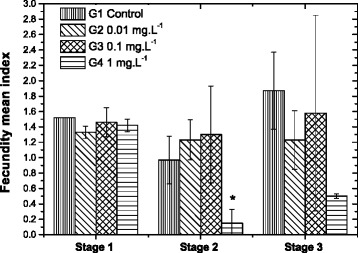
**Mean fecundity index of** ***G. tigrina*** **planarian exposed to commercial textile dye disperse red 1 (control and exposed groups).** At stage 1, exposure system adaptation, organisms were monitored for 2 weeks in maintenance medium. At stage 2, organisms were exposed for 5 weeks. At stage 3, recovery, exposed organisms were monitored for 2 weeks more in maintenance water. Bars represent the standard deviation of the mean. *Significant at p ≤ 0.05.

Freshwater planarian reproduction is a complex process and some details are still unknown [22]. These animals have a many-sided reproductive organ with ovaries and hormones. Animals can be fertilized after mating [23,46]. Eggs, formed after fertilization, as well as vitelline cells, are enveloped by an oval wall called cocoons [47]. Hatching occurs after different incubation periods depending on the species and temperature [40,48]. The time between crosses and cocoon lay is still unknown. In our experiments, the fecundity index recorded for the animals exposed to 1 mg/L of disperse red 1 (stage 2, exposure) was statistically significantly lower than the control (Figure 3). Also, after the first week of exposure, animals from the treated group G4 (1 mg/L) presented colored skin and alterations in behavior during feeding, such as continuous exposure of the pharynx and limited mobility. The pharynx is only exposed during a planarian's feeding process, and after feeding this organ returns to planarian's gastric cavity [40]. However, we observed that planarians, when exposed to the dye, showed retractile pharynx movements even when they were far from the food. Planarian reproductive success is related to food availability, demographic density, and temperature [22,48,49]. During the entire exposure time, the animals from G4 group were not able to eat and the cocoons produced during that time may have been generated using their remaining energy [36,50]

During stages 1 (before exposure) and 3 (recovery following exposure) no statistical differences were observed in the fecundity indexes among the groups, although fecundity capacity did not seem to be fully recovered when animals were exposed to 1 mg/L during stage 3 (Figure 3). The addition of the recovery stage was also useful to analyze if the planarians were able to remove the impregnated or absorbed dye from their bodies and if the effect on the pharynx's retractability was permanent. After 2 weeks of recovering in maintenance water more than 80% of the exposed animals showed normal skin color as well as normal pharynx function and feeding behavior. Therefore, disperse red 1 interferes with *G. tigrina*'s mobility and capacity for food identification at 1 mg/L exposure concentration. In sum, disperse red 1 dye adversely affects survival, fecundity, regeneration, and feeding behavior in planarians. Based on the most critical endpoint (fecundity index) it is possible to suggest a no-observed-adverse-effect-concentration (NOAEC) of 0.1 mg/L for disperse red 1. As observed in other studies [15,17,21,22,49], the viability of *G. tigrina* culture under low-cost laboratorial conditions was confirmed. Our results also show the repeatability of the applied protocol at least for zinc and disperse red 1.

Planarians can be considered an interesting model for ecotoxicological assessment of environmental contaminants because it allows the assessment of different sub-lethal effects, such as increase in mucus production and changes in behavior besides the evaluation of adverse effects on the regeneration process and reproduction as showed in this work.

## Conclusions

Disperse red 1 presented a median LC_50_ of 75 and 152 mg/L, respectively, for newborns and adults of *G. tigrina*, showing that newborns are more susceptible to the dye than adults. Uncoordinated movements, irregular twists, colored skin, and increased mucous production were observed after dye exposure at sub-lethal concentrations. A no-observed-adverse-effect concentration (NOAEC) of 0.1 mg/L of disperse red 1 could be determined based on the fecundity test. Zinc seems to be a suitable reference substance for monitoring the sensitivity in *G. tigrina* tests within only 24 h of exposure. This study successfully demonstrates the applicability of *G. tigrina* tests in the hazard evaluation of azo dyes, a relevant example of an emerging contaminant. The results obtained in this work may also be used for the determination of safe environmental concentrations of disperse red 1 for the aquatic system.

## Methods

### Chemical

The powdered commercial textile dye product disperse red 1 dye (*N*-Ethyl-*N*-(2-hydroxyethyl)-4-(4-nitrophenylazo) aniline; CAS No 2872-52-8), was purchased from PCIL© Dyes, São Paulo, Brazil. The product was chemically characterized by Vacchi et al. [11] and contains 60% of the main dye disperse red 1, 20% of other azo dye products, and 20% of a surfactant. Zinc sulfate (ZnSO_4_.7H_2_O; CAS No 7440-20-0; CAQ Casa de Química©, Diadema, São Paulo, Brazil) and potassium dichromate (K_2_Cr_2_O_7_; CAS No 7778-50-9; Cromoline Química Fina©, Diadema, São Paulo, Brazil) in analytical grade were used as reference substances to monitor the sensitivity of the planarian culture. Dye and metal testing solutions were prepared in planarian maintenance water (see description below) in concentrations determined by preliminary tests.

### Animals

Specimens of *G. tigrina* collected in a pristine pond in Rio Claro, São Paulo, Brazil [17], have been cultivated in the Ecotoxicology and Environmental Microbiology Laboratory (LEAL) in the University of Campinas, Limeira, Brazil, since 2005. Descendant animals were cultured by sexual reproduction at room temperature (22 to 25°C) under a light/dark photoperiod of 16:8 h, and fed once a week with bovine liver. The water for culturing the planarians and conducting experiments was designated as maintenance water and was prepared with dechlorinated tap water and 45 mg CaCO_3_/L, pH 7.6 ± 0.3, and with gentle aeration [37] using an aquarium pump. Water was completely renewed each week after feeding.

### Toxicity tests

Three environmentally relevant endpoints were investigated in this study through different toxicity tests: acute toxicity (mortality), regeneration assay and reproductive performance. Acute toxicity tests were carried out with adult and newborn planarians. Animals of more than 3 months of age and visible cocoon production capacity were considered adults, and animals with a maximum age of 10 days were considered newborns [15]. Each independent newborn toxicity test was carried out in duplicate using seven animals placed in 50 mL of test solution for 96 h [15]. In the adult toxicity tests healthy animals were randomly selected and five planarians were exposed to 100 mL of test solution. Each independent experiment was performed in triplicate [37]. In both the newborn and adult tests, the test solution was not renewed and the animals were not fed. Negative controls with maintenance water and positive controls with ZnSO_4_ and K_2_Cr_2_O_7_ were also performed. The nominal concentration used in the positive controls using chrome were 0.9, 1.8, 3.5, 7, 10, 18 and 35 mg/L expressed as Cr^6+^ and using zinc were 0.1, 0.2, 0.5, 1.1, 2.2, 4.5, and 6.8 mg/L, expressed as Zn^2+^. Mortality, body degeneration, change in behavior and morphological alterations were monitored every 24 hours under a stereo-microscope (Stemi 2000-C, Zeiss©, Oberkochen, Germany). The nominal dye concentrations tested were 10, 50, 100, 150, and 200 mg/L for newborns and 50, 100, 150, 200, and 250 mg/L for adults.

For the regeneration assay, the heads of adult planarians were removed in the region behind the auricles using blades, and the decapitated animals were used immediately [14]. The animals were fed 1 day before the test started to ensure that they would have sufficient nutrients to regenerate. This is important because *G. tigrina* has the ability to store vital nutrients [36] and the digestion and assimilation of food, in particular liver, takes about 197 h [50]. The concentrations of disperse red 1 used (10, 50, 100, 150 and 200 mg/L) were selected based on the toxicity test results. In each independent experiment, each concentration was tested using four replicates; five decapitated animals per replicate were put in 20 mL of solution. Each head regeneration step [17,31] (designated, a decapitated bodies; b beginning of blastema formation; c regular blastema; d beginning of auricle formation; e beginning of eyespot formation; f auricle and eyespots clearly defined; g full head formation; and t body section without cicatrization, z without body cicatrization, with eyespots, and without auricle) were observed under the stereoscope daily, followed by renewal of the dye test solution [20]. Regeneration of decapitated planarians in maintenance water was used as a negative control. Animals were not fed, and they were kept in cultivation conditions for 120 h for full regeneration of the encephalic region [17].

The reproductive performance of adult specimens was evaluated based on a method described elsewhere [20]. The single experiment included a preexposure stage for adaptation purposes. We also followed the recovery capacity in a postexposure period, without the presence of the toxicant. Twenty-five healthy adult animals were randomly placed into plastic vessels containing 1 L of each test solution, without replicates. The test was carried out in three stages: at stage 1 (adaptation), all organisms were exposed only to maintenance medium and were monitored for 2 weeks. At stage 2 (toxicant exposure), three groups of organisms were exposed to sub-lethal concentrations (0.01, 0.1, or 1 mg/L) of the commercial product. We also included a negative control, without the dye. Concentrations were selected based on the acute toxicity test results. This stage lasted five weeks. At stage 3 (recovery), chemical exposure was interrupted at the end of the 5 weeks; animals were transferred into maintenance water and monitored for an additional 2 weeks. Observations on cocoon production, animal behavior, and mortality were made during all stages. Identical cultivation maintenance procedures were used for all groups, with weekly feeding followed by total test solution renewal, and the counting and segregation of cocoons.

During the toxicity, reproductive, and regeneration experiments, any changes in animal behavior, skin color, and mucous production were registered on a daily basis. The retractability of the pharynx during feeding was also monitored.

### Statistical analysis and expression of the results

For acute toxicity tests, the trimmed Spearman-Karber method [51] was used to calculate the LC_50_, with 95% confidence interval, for every 24 h of exposure, until 96 h. In the regeneration tests, a 120-h delayed regeneration value (Pd120h) was calculated for each concentration. To obtain this value, the number of delayed regeneration planarians in the control was subtracted from the treated number. The NOAEC was the higher concentration that presented a Pd120h <1, and the LOAEC was the lowest concentration that presented a Pd120h ≥1.

In the reproductive performance assay, the mean fecundity index for each group in each stage was calculated using a method adapted from Knakievicz et al. [22]. Mean fecundity indexes were calculated by the total number of cocoons divided by the number of live planarians in every analyzed week, divided by the total experimental time, in weeks, at the end of each step. To compare the different treatments, analysis of variance (ANOVA) followed by the Tukey test was performed using the Origin® Pro8 program. The significance level was *p* ≤ 0.05.

## Abbreviations

LC_50_: median lethal concentration
EC_50_: median effective concentration
GHS: Global Harmonization System
NOAEC: no-observed-adverse-effect concentration
LOAEC: lowest-observed-adverse-effect concentration
LEAL: Ecotoxicology and Environmental Microbiology Laboratory
CAS: Chemical Abstracts Service
Pd: delayed regeneration value
ANOVA: analysis of variance
